# Detailed Dissection of UBE3A-Mediated DDI1 Ubiquitination

**DOI:** 10.3389/fphys.2019.00534

**Published:** 2019-05-03

**Authors:** Nagore Elu, Nerea Osinalde, Javier Beaskoetxea, Juanma Ramirez, Benoit Lectez, Kerman Aloria, Jose Antonio Rodriguez, Jesus M. Arizmendi, Ugo Mayor

**Affiliations:** ^1^Department of Biochemistry and Molecular Biology, Faculty of Science and Technology, University of the Basque Country (UPV/EHU), Leioa, Spain; ^2^Department of Biochemistry and Molecular Biology, Faculty of Pharmacy, University of the Basque Country (UPV/EHU), Vitoria-Gasteiz, Spain; ^3^Proteomics Core Facility-SGIKER, University of the Basque Country (UPV/EHU), Leioa, Spain; ^4^Department of Genetics, Physical Anthropology and Animal Physiology, University of the Basque Country (UPV/EHU), Leioa, Spain; ^5^Ikerbasque – Basque Foundation for Science, Bilbao, Spain

**Keywords:** UBE3A, ubiquitin E3 ligase, Angelman syndrome, proteasome, mass-spectrometry, GFP pull-down

## Abstract

The ubiquitin E3 ligase UBE3A has been widely reported to interact with the proteasome, but it is still unclear how this enzyme regulates by ubiquitination the different proteasomal subunits. The proteasome receptor DDI1 has been identified both in *Drosophila* photoreceptor neurons and in human neuroblastoma cells in culture as a direct substrate of UBE3A. Here, we further characterize this regulation, by identifying the UBE3A-dependent ubiquitination sites and ubiquitin chains formed on DDI1. Additionally, we found one deubiquitinating enzyme that is capable of reversing the action of UBE3A on DDI1. The complete characterization of the ubiquitination pathway of an UBE3A substrate is important due to the role of this E3 ligase in rare neurological disorders as Angelman syndrome.

## Introduction

The lack of functional UBE3A E3 ubiquitin ligase in the brain is responsible for a rare neurodevelopmental disorder called Angelman syndrome (AS). With an incidence of 1:15.000 births (Margolis et al., 2015), the disease is characterized by episodes of frequent laughter, language impairment, severe developmental delay, ataxic movements, hypopigmentation, seizure disorder, sleep disturbances, muscular hypotonia, and motor delay (Buiting et al., 2016). Clinical features of AS were first described in 1965, and since then, the diagnosis criteria has been well established (Williams et al., 2006). Moreover, some studies established a link between the affected molecular mechanisms and their consecutive symptoms in patients. For example, seizure disorders have been associated to haploinsufficiency of GABA receptor genes (Moncla et al., 1999) and hypopigmentation of the skin has been explained by UBE3A regulating the melanocortin 1 receptor (Low and Chen, 2011). However, the role of UBE3A on the brain and how the lack of it can cause such a severe clinical scenario is still unknown.

Protein ubiquitination involves the coordinated action of three complementary enzymes. Upon E1-mediated activation, ubiquitin (Ub) is transferred to an E2 ubiquitin-conjugating enzyme, and subsequently to an E3 ubiquitin ligase, which is the ultimate responsible for attaching the ubiquitin moiety to the substrate (Glickman and Ciechanover, 2002). UBE3A is a HECT-type E3 ligase that catalyzes Ub transfer to the substrate through a two-step reaction that involves first, the transfer of Ub to a catalytic cysteine on the E3, and then, from the E3 to the substrate (Glickman and Ciechanover, 2002). Ub is usually attached to the side chain of lysine residues, and thus, any lysine-containing protein could potentially be modified with ubiquitin. Such modification may occur as a single Ub attached to a single lysine (mono-ubiquitination) or to several lysines (multiple mono-ubiquitination). In addition, ubiquitin itself contains eight available amide groups: 7 lysines (K6, K11, K27, K29, K33, K48, K63) and a *N*-terminal amide group (M1) by which additional ubiquitin moieties can be attached, forming a wide range of poly-ubiquitin chains (Komander and Rape, 2012). Linkages in a poly-ubiquitin chain can be mediated by the same lysines across the chain or by different lysines, which results in the formation of homogenous and heterogeneous chain types, respectively. Additionally, if multiple lysines in each Ub are involved in the linkage formation, branched chains are formed.

Different ubiquitin chain types assign proteins a great variety of functions (Swatek and Komander, 2016). Mono-ubiquitination is involved in many essential cellular roles, including receptor internalization (Haglund et al., 2003), subcellular localization (Landré et al., 2017) and regulation of transcriptional activity (Pham and Sauer, 2000). Among poly-ubiquitin chains, K48 chains are the best characterized and are typically associated to protein degradation (Glickman and Ciechanover, 2002; Ciechanover, 2013). Indeed, *in vitro* studies have demonstrated that UBE3A ubiquitin ligase mainly forms K48 chains and hence, likely targets proteins to the proteasome (Wang and Pickart, 2005; Kim and Huibregtse, 2009). Nevertheless, poly-ubiquitin chains also exert a number of non-degradative roles. It has recently been shown that K11/K48-linked ubiquitin chains play a key role in cell cycle and quality control (Yau et al., 2017). Additionally, K63-linked poly-ubiquitination is required for the cytoplasmic localization of MBNL1 (Wang et al., 2018). K29 poly-ubiqutination is a negative regulator of Wnt/β-catenin signaling (Fei et al., 2013), whereas M1, K11 and M1/K63 mixed Ub chains modulate the NF-κb signaling pathways (Tokunaga et al., 2009; Dynek et al., 2010; Yau et al., 2017). There is also evidence for the involvement of K6-, K27- and K33-linked ubiquitination in the DNA damage response (Elia et al., 2015). Altogether, it is evident that in order to characterize the role of ubiquitination in the regulation of a given protein, it is essential to first identify the types of ubiquitin chain linkages that are formed on it.

Since ubiquitination controls the diverse endpoints of proteins, in Angelman syndrome patients, UBE3A substrates are likely to be negatively affected by the lack of functional UBE3A in neurons. In order to better understand the molecular mechanisms involved in this disease, it is pivotal not only to identify the neuronal substrates of UBE3A *in vivo*, but also to characterize their ubiquitin chains. In previous *in vivo* studies using flies, we searched for UBE3A substrates (Ramirez et al., 2018), and noted that the ubiquitination levels of many proteasomal subunits were significantly enhanced upon UBE3A overexpression. In agreement with other studies (Jacobson et al., 2014; Tomaić and Banks, 2015; Yi et al., 2017), this leads to the idea that UBE3A could regulate the activity of the proteasome. In this regard, we confirmed that the proteasomal shuttling protein Rngo/DDI1, which itself targets poly-ubiquitinated proteins to proteasomal degradation (Saeki et al., 2002; Kaplun et al., 2005; Ivantsiv et al., 2006; Voloshin et al., 2012; Ramirez et al., 2018), is a direct substrate of UBE3A (Ramirez et al., 2018). Nevertheless, it remains to be elucidated how UBE3A modulates the ubiquitination pattern of DDI1, and how this modification affects DDI1.

Overall, protein ubiquitination is not only modulated by E3 ligases, but also by deubiquitinating (DUB) enzymes that are responsible for removing the ubiquitin moiety from substrates. The human genome codes for almost a hundred DUBs that based on sequence similarity and likely mechanisms of action are divided into six groups: UCH, USP, OTU, JAMM, MJD, and the most recently discovered MINDY (Amerik and Hochstrasser, 2004; Abdul Rehman et al., 2016). It could be anticipated that there might be at least one specific DUB that counteracts the action of UBE3A. Numerous studies have shown the great potential of DUBs as suitable drug targets to treat cancer, neurodegenerative diseases and viral infection (Edelmann et al., 2011; Huang and Dixit, 2016). Therefore, identifying the DUB responsible for deubiquitinating UBE3A substrates is of pivotal relevance in the development of successful therapies to treat Angelman syndrome. More precisely, pharmacological inhibition of such DUB might help recovering the non-pathological condition of those patients, recovering to some degree the ubiquitination of those substrates shared with UBE3A.

In the present study, we have characterized the UBE3A-dependent ubiquitination of the proteasomal shuttling protein DDI1. From six ubiquitination sites detected on DDI1, we have discovered that the presence of K133 is necessary for DDI1 to be ubiquitinated by UBE3A. Additionally, investigation of the ubiquitin linkages has shown that UBE3A forms K11- and K48-linked ubiquitin chains on DDI1. We also screened a siRNA library to search for the DUB involved in the deubiquitination of UBE3A substrates, and found that USP9X has the capacity to regulate DDI1 ubiquitination levels. Overall, our data shed light into the molecular mechanisms underlying Angelman syndrome, and reveal USP9X as a potential therapeutic target that may help restoring the non-pathological ubiquitination pattern on Angelman syndrome patients, and hence, ameliorate their symptoms.

## Materials and Methods

### Plasmids

*C*-terminally GFP-tagged DDI1 (DDI1-GFP) vector was generated using the commercial pEGFP-N1 vector (Clontech), while *N*-terminally GFP-tagged DDI1 (GFP-DDI1) was cloned into a PM-cherry-C1 vector kindly provided by Dr. Daniel Abankwa (see section “Cloning Procedures”). The plasmids used to express the *N*-terminally FLAG-tagged versions of the wild type (FLAG-UBE3A-pCMV, UBE3A^WT^) and catalytically inactive (FLAG-UBE3A^LD^-pCMV, UBE3A^LD^) human UBE3A (Tomaić et al., 2009) were a gift from Dr. Vjekoslav Tomaic. FLAG-tagged ubiquitin (FLAG-Ub) cloned in pCDNA3.1 vector (Invitrogen) was used (Ramirez et al., 2018). A previously described untagged human Parkin plasmid (Martinez et al., 2017) was used and the empty pCDNA3.1 vector (Invitrogen) was used as control (Ramirez et al., 2018).

### Cloning Procedures

Gene for human DDI1 protein (Uniprot Q8WTU0) was synthesized by GenScript and further amplified using 5′-TATAGGTACCATGCTGATCACCGTG-3′ forward primer and 5′-TATAACCGGTATGCTCTTTTCGTCC-3′ reverse primer and inserted between the Acc65I and AgeI sites of the pEGFPN1 vector. For the generation of GFP-DDI1, human DDI1 sequence was amplified using 5′-CGCGTGTACAGTATGAATATAGCGATA-3′ forward primer and 5′-CGCAAGCTTTCAATGCTCTTTTCG-3′ reverse primer. Amplified DDI1 was then inserted between BsrGI and HindIII sites of the PM-cherry-C1 where mCherry had been previously replaced by GFP. All PCR reactions were carried out with Phusion High-Fidelity DNA polymerase (Thermo Scientific). PCR product gel extractions and plasmid purifications were performed with the E.Z.N.A. Gel Extraction Kit and E.Z.N.A. plasmid mini and midi kits (Omega Bio-tek), respectively. Correct sequence for all plasmids was confirmed by sequencing either by the Eurofins GATC Biotech Company (Köln, Germany) or the SGIKER Unit of Sequencing and Genotyping at the University of the Basque Country (Leioa, Spain).

### Cell Culture and Transfection

Human Embryonic Kidney cells (HEK293T) were cultured under standard conditions (37°C, 5% CO2) using Dulbecco’s modified Eagle medium/nutrient mixture F-12 (DMEM/F-12) with GlutaMAX (Thermo Scientific) and supplemented with 10% fetal bovine serum (Thermo Scientific), 100 U/ml of penicillin (Invitrogen) and 100 mg of streptomycin (Invitrogen). For ubiquitination sites and chain-types experiments by mass spectrometry, HEK293T cells were seeded in 100 mm plates (2 × 10^6^ cells). After 24 h from seeding, cells were co-transfected with 12,5 μg of DDI1-GFP and 12,5 μg of UBE3A^WT^ or UBE3A^LD^ for 72 h using Lipofectamine 3000 transfection reagent (Invitrogen) and according to manufacturer’s instructions. For the DUB silencing experiment, HEK293T cells were seeded the previous day in six-well plates (6 × 10^5^ cells). Each well was then transfected with 10 nM siRNA targeting specific DUBs (Ambion; life technologies). For that purpose Lipofectamine RNAiMAX transfection reagent (Thermo Fischer) was used according to manufacturer’s instructions. After 24 h incubation, 1,5 μg of DDI1-GFP and 1,5 μg of FLAG-Ub were co-transfected for another 48 h using again Lipofectamine 3000 transfection reagent (Invitrogen). For all experiments, cells were washed twice in PBS after the transfection periods and stored at -20°C until required.

### GFP Pull-Down Protocol

Transfected HEK293T cells were lysed with 500 μl of lysis buffer in six-well plates and with 2 mL in 100 mm dishes (50 mM Tris–HCl pH 7.5, 150 mM NaCl, 1 mM EDTA, 0.5% Triton, 1× protease inhibitor cocktail from Roche Applied Science and 50 mM *N*-ethylmaleimide from Sigma) and centrifuged at 14 000 *g* for 10 min. Supernatants were mixed with 25 μl/well (6-well plate) and 50 μL/well (100 mm plates) of GFPTrap-A agarose beads suspension (Chromotek GmbH), which had been previously washed twice with a Dilution buffer (10 mM Tris–HCl pH 7.5, 150 mM NaCl, 0.5 mM EDTA, 1× protease inhibitor cocktail, 50 mM *N*-ethylmaleimide). After incubation for 3 h at room temperature with gentle rolling, samples were centrifuged at 2700 *g* for 2 min to separate the beads from the unbound material. GFP beads were then subjected to three washing steps; once with the dilution buffer, thrice with washing buffer (8 M urea, 1% SDS in PBS) and once with 1% SDS in PBS. GFP-tagged proteins bound to the beads were eluted by incubating at 95°C for 10 min with 25 μl elution buffer (250 mM Tris–HCl pH 7.5, 40% glycerol, 4% SDS, 0.2% bromophenol blue, 100 mM DTT).

### Western Blotting and Silver Staining

For SDS–PAGE, 4–12% Bolt Bis–Tris Plus pre-cast gels (Invitrogen) were used. Proteins were then transferred to PVDF membranes using the iBlot system (Invitrogen). Membranes were blocked using 5% powdered milk in PBS-Tween (PBS-T). Following primary and secondary antibody incubation, membranes were developed with an ECL kit (Biorad Clarity). To create dual-color westerns independent color channels were assigned to two independent westerns that were developed in the same membrane. The amount of material loaded for western blot analysis was between 10–30% of neat inputs and 30–50% of neat elution samples. The following primary antibodies were used at 1/1000 concentration: mouse monoclonal anti-GFP antibody (Roche Applied Science; catalog number 11814460001); mouse monoclonal anti-FLAG M2-HRP conjugated antibody (Sigma; catalog number A8592); mouse monoclonal anti-UBE3A (clone E6AP-300) antibody (Sigma; catalog number E8655), rabbit monoclonal anti-UCHL-5 (Abcam, ab124931), rabbit polyclonal anti-USP9X (Proteintech, 55054-1-AP), anti-USP7 (kindly provided by Dr. Emilio Lecona), rabbit polyclonal anti-USP42 (Sigma, HPA006752). The following secondary antibodies were used at 1/4000 concentration: goat anti-mouse-HRP-labeled antibody (Thermo Scientific; catalog number 62-6520) and goat anti-rabbit-HRP labeled antibody (Cell Signaling; catalog number 7074).

Prior to MS analysis, the efficiency of GFP pull-downs was evaluated by silver staining. Briefly, the 10% of each neat elution was resolved by SDS–PAGE (4–12% Bolt Bis–Tris Plus precast gels, Invitrogen), and after fixing the proteins within the gel with 40% methanol/10% acetic acid, gel bands were visualized using the SilverQuest kit following manufacturer’s instructions (Invitrogen).

### In-Gel Trypsin Digestion and Peptide Extraction

**FIGURE 1:**
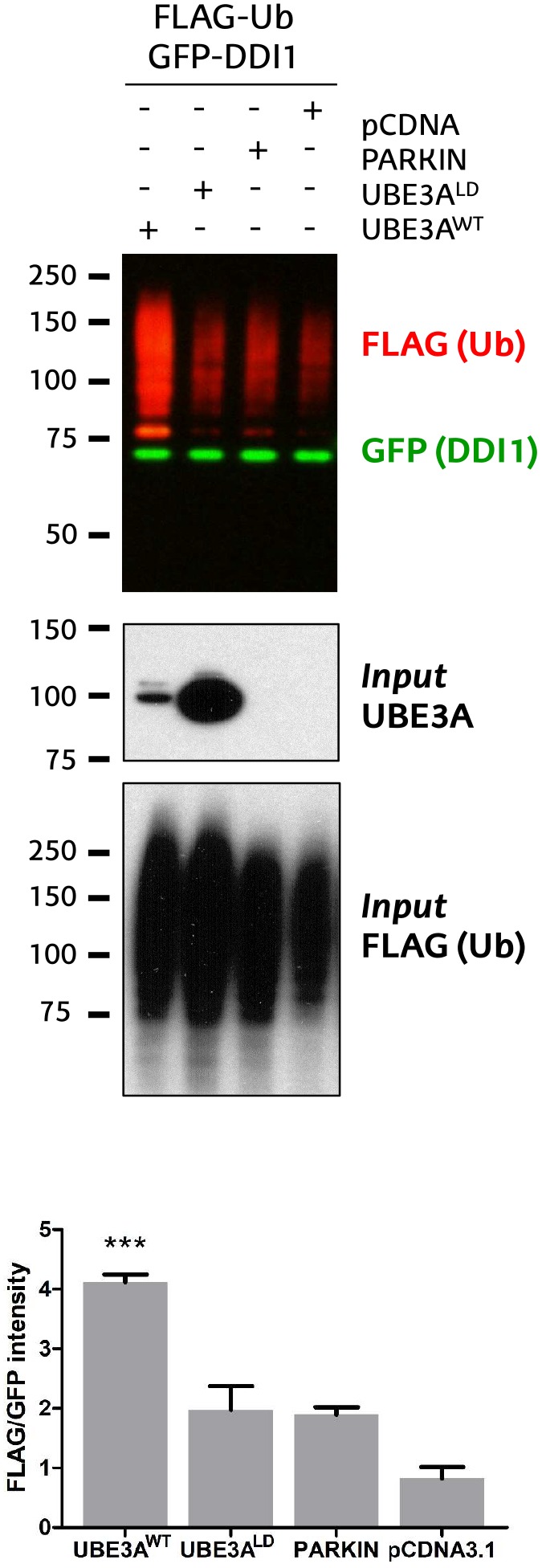
Validation of DDI1 as a substrate of UBE3A in HEK293T cells. DDI1 ubiquitination was detected by western blot upon overexpression of wild-type UBE3A (UBE3A^WT^), ligase dead UBE3A (UBE3A^LD^), Parkin E3 ligase (PARKIN) and control vector (pCDNA3.1) in HEK293T cells. Anti-FLAG antibody (red) was used to detect the ectopically expressed ubiquitin, whereas the non-modified GFP-tagged DDI1 was detected by anti-GFP antibody (green). UBE3A overexpression was confirmed in whole cell lysates using anti-UBE3A antibody (*Input* UBE3A) while the FLAG signal in the inputs corroborated equivalent transfections in all samples [Input FLAG-(Ub)]. Quantification of western blots was performed with Image-J, by normalizing the FLAG intensities to the GFP signal. The analysis showed a statistically significant increase [one-way ANOVA, ^∗∗∗^*p*-value < 0.0001, (mean ± S.E.M., *n* = 3)] of GFP-DDI1 ubiquitination upon UBE3A^WT^ overexpression in comparison to overexpression of UBE3A^LD^.

Ninety percent of the neat elution from each GFP pull-down was resolved by SDS–PAGE (4–12% Bolt Bis–Tris Plus precast gels, Invitrogen) and visualized with Colloidal Blue (Invitrogen) following manufacturer’s instructions. Aiming to isolate mono- and poly-ubiquitinated DDI1, each gel lane was excised into two slices above the clear band of approximately 75 KDa that corresponds to non-modified DDI1 (Figure 1). Subsequently, each gel lane was chopped, and proteins were subjected to in-gel digestion protocol using trypsin as described before (Osinalde et al., 2015). Briefly, gel pieces, previously dehydrated with acetonitrile (ACN), were reduced with 10 mM dithiothreitol (DTT), alkylated with 55 mM chloroacetamide, and finally rehydratated in 12.5 ng/ml trypsin and incubated overnight at 37°C. The following day, resulting tryptic peptides were extracted from the gel by serial incubation with 100% ACN and 1% trifluoroacetic acid in 30% ACN. Finally, prior to MS analysis, peptide solutions were dried down in a SpeedVac centrifuge (Thermo Scientific).

### LC-MS/MS Analysis

Mass spectrometric analyses were performed on an EASY-nLC 1200 liquid chromatography system interfaced with a Q Exactive HF-X mass spectrometer (Thermo Scientific) via a nanospray flex ion source. Dried peptides were dissolved in 0.1% formic acid and loaded onto an Acclaim PepMap100 pre-column (75 μm × 2 cm, Thermo Scientific) connected to an Acclaim PepMap RSLC C18 (75 μm × 25 cm, Thermo Scientific) analytical column. Peptides were eluted from the column using the following gradient: 18 min from 2.4 to 24%, 2 min from 24 to 32% and 12 min at 80% of acetonitrile in 0.1% formic acid at a flow rate of 300 nl min^-1^. The mass spectrometer was operated in positive ion mode. Full MS scans were acquired from m/z 375 to 1800 with a resolution of 120,000 at m/z 200. The 10 most intense ions were fragmented by higher energy *C*-trap dissociation with normalized collision energy of 28 and MS/MS spectra were recorded with a resolution of 15,000 at m/z 200. The maximum ion injection time were 100 ms and 120 ms, whereas AGC target values were 3 × 10^6^ and 5 × 10^5^ for survey and MS/MS scans, respectively. In order to avoid repeat sequencing of peptides, dynamic exclusion was applied for 12 s. Singly charged ions or ions with unassigned charge state were also excluded from MS/MS. Data were acquired using Xcalibur software (Thermo Scientific).

### Data Processing and Bioinformatics Analysis

Acquired raw data files were processed with the MaxQuant (Cox and Mann, 2008) software (version 1.5.3.17) using the internal search engine Andromeda (Cox et al., 2011) and searched against the UniProt database restricted to *Homo sapiens* entries (release 2017_03; 42165 entries) where the sequences of FLAG_UBIQUITIN and DDI1_GFP proteins were manually added. To assess ubiquitin chains types present on DDI1, only raw files corresponding to poly-ubiquitinated DDI1 were processed. By contrast, to infer the DDI1 ubiquitination sites, raw files corresponding to both mono- and poly-ubiquitinated DDI1 were processed together. Carbamidomethylation (C) was set as fixed modification whereas Met oxidation, protein *N*-terminal acetylation and Lys GlyGly (not *C*-term) were defined as variable modifications. Mass tolerance was set to 8 and 20 ppm at the MS and MS/MS level, respectively. Enzyme specificity was set to trypsin, allowing for a maximum of three missed cleavages. Match between runs option was enabled with 1.5 min match time window and 20 min alignment window to match identification across samples. The minimum peptide length was set to seven amino acids. The false discovery rate for peptides and proteins was set to 1%. For the analysis of ubiquitination sites, a minimum localization probability of 0.75 was used. Normalized spectral protein label-free quantification (LFQ) intensities were calculated using the MaxLFQ algorithm.

## Results

### UBE3A-Dependent Ubiquitination Sites on DDI1

We recently discovered that Ube3a ubiquitinates endogenous Rngo in flies, and also its human homolog DDI1 when transfected in S5-SYHY neuroblastoma cells (Ramirez et al., 2018). Using a GFP-based pulldown protocol (Ramirez et al., 2016), we now confirm that UBE3A ubiquitinates also DDI1 in HEK293T kidney cells. As compared to co-transfection with an empty vector (pCDNA3.1), or another E3 ligase such as PARKIN, a ligase dead version of UBE3A (UBE3A^LD^) does not increase the ubiquitination of GFP-tagged DDI1. By contrast, wild type UBE3A (UBE3A^WT^) enhances the ubiquitination of DDI1 while the level of non-modified DDI1 remains unchanged (Figure 1 and Supplementary Figure 1). This effect is independent of whether DDI1 is *N*- or *C*-terminally fused to GFP (Supplementary Figure 2). Interestingly, UBE3A-mediated ubiquitination of DDI1 does not seem to target it for degradation, as total GFP-tagged DDI1 levels are equivalent in all samples.

**FIGURE 2:**
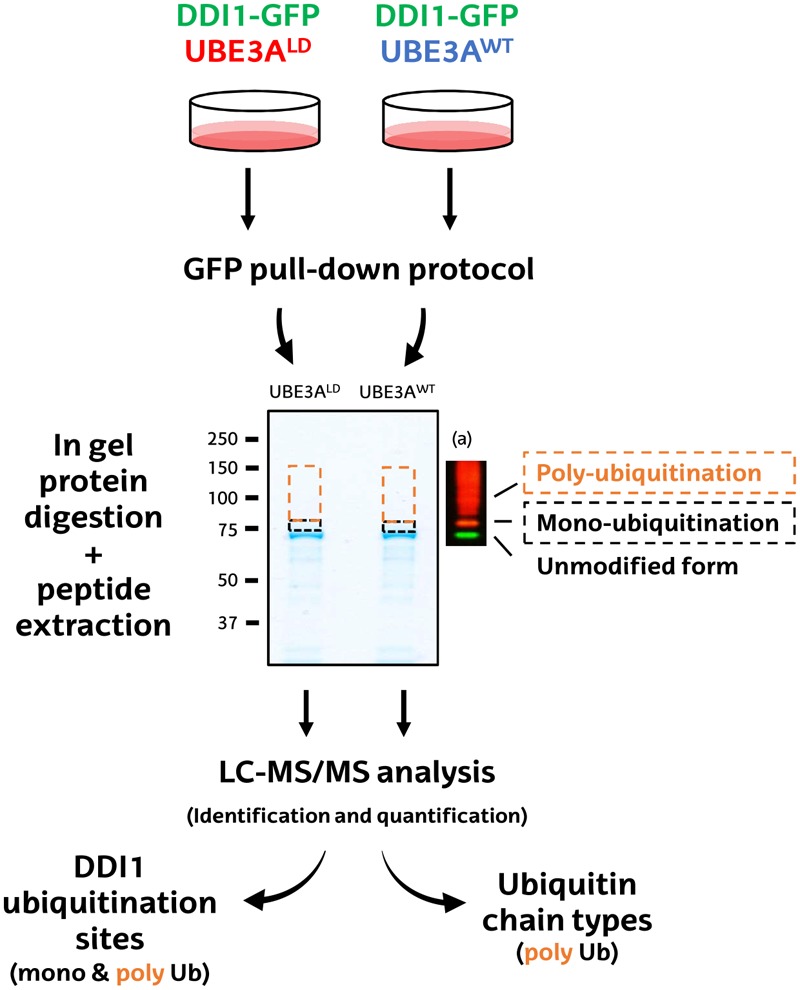
Schematic diagram of the MS-based procedure used to study ubiquitination sites and ubiquitin chain types on DDI1. HEK293T cells were co-transfected with DDI1-GFP and wild type UBE3A (UBE3A^WT^) or ligase dead UBE3A (UBE3A^LD^). Unmodified DDI1-GFP as well as its ubiquitinated forms were isolated by GFP pull-down, and separated by SDS–PAGE. The gel slice corresponding to ubiquitinated DDI1-GFP was excised and divided into two, basing on a previous western blot (a); a lower band corresponding for mono-ubiquitinated DDI1-GFP (black), and a band above it corresponding to poly-ubiquitinated DDI1-GFP (orange). Gel slices were digested with trypsin, and resulting peptides were extracted from the gel to further identify and quantify them by LC-MS/MS. Both bands were analyzed to infer DDI1 ubiquitination sites, whereas only the poly-ubiquitination band was used to detect the nature of the ubiquitin chain types formed on DDI1.

According to PhosphositePlus database – the most comprehensive repository of PTM sites – (Hornbeck et al., 2015), human DDI1 can be ubiquitinated on K345 and K346. Nevertheless, DDI1 contains twelve additional lysines that may potentially be modified by ubiquitin. With the aim to identify the ubiquitination sites on DDI1 that are modified by UBE3A, we followed an approach that optimized the GFP pull-down protocol (Ramirez et al., 2016) for mass spectrometry (MS)-based detection (Figure 2).

First, GFP-tagged DDI1 was transfected on HEK293T cells together with wild type or, as control, ligase dead UBE3A (UBE3A^WT^ and UBE3A^LD^, respectively). Then, DDI1 was isolated from both experimental conditions by GFP pull-down procedure, and subsequently resolved by SDS–PAGE. Coomassie staining revealed a band just below 75 kDa corresponding to GFP-tagged DDI1, as earlier identified by western blotting (Figure 1). As we were interested in the ubiquitinated fraction of DDI1, we excised a slice of the gel directly above that band (Figure 2). Aiming to separate mono- and poly-ubiquitinated DDI1, this gel piece was further divided into two subsequent slices based on the observations by silver staining (Supplementary Figure 3) as well as by previous immunoblotting results (Figure 1): a tight band just above the non-modified DDI1 should correspond to mono-ubiquitinated DDI1-GFP and a bigger slice above this one, corresponding to the smear of poly-ubiquitinated DDI1 observed by immunobloting. Then, proteins within each gel slice were subjected to trypsin digestion, resulting peptides were extracted from the gel, and analyzed by MS; this protocol from the transfection of cells to the MS analysis was performed in triplicate for each experimental sample.

**FIGURE 3:**
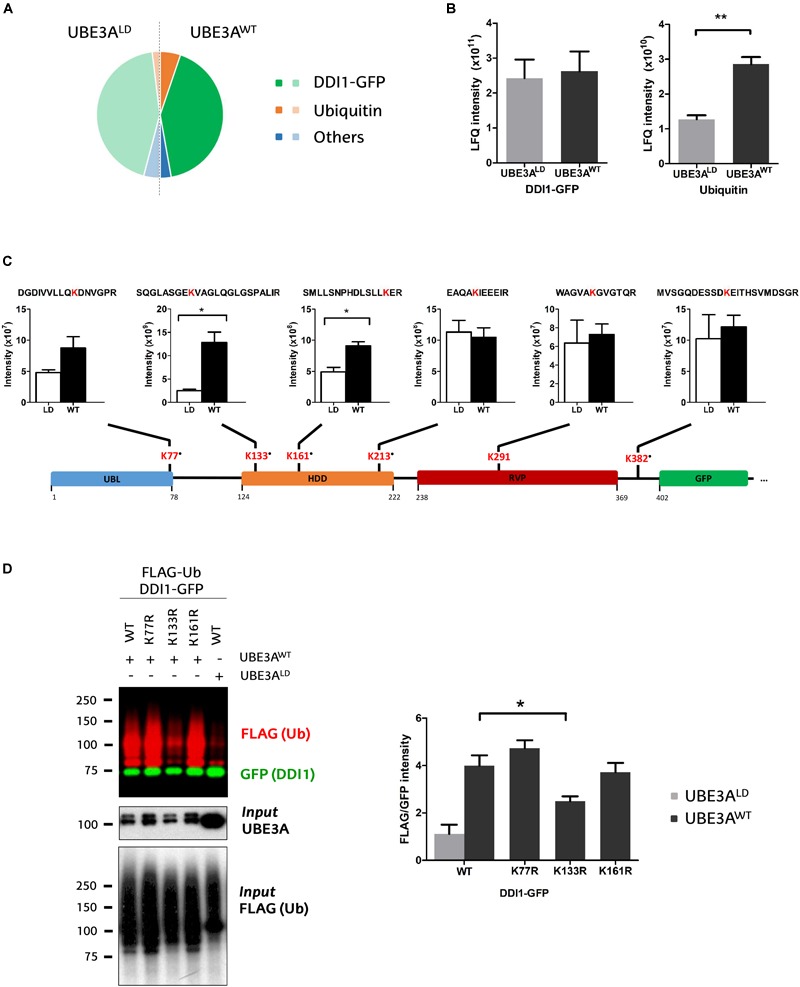
Identification and quantification of DDI1 ubiquitination sites. **(A)** Relative LFQ intensity of all the proteins detected by MS demonstrate that DDI1-GFP is the most abundant protein (green) in the GFP pull-down samples, followed by ubiquitin (orange). 287 more proteins (blue) were identified with marginal intensities. **(B)** Comparison of the intensities recorded for the two most abundant proteins in UBE3A^WT^ and UBE3A^LD^ reveal that whereas the levels of DDI1 detected are similar in both conditions, more ubiquitin was detected in the presence of UBE3A^WT^ overexpression [*t*-test, ^∗∗^*p*-value < 0.05, (mean ± S.E.M., *n* = 3)]. **(C)** Schematic illustration of DDI1-GFP sequence, its domains (blue, UBL, ubiquitin-like domain; orange, HDD, helical-double domain; red, RVP, retroviral protease domain; green, GFP, GFP-tag of DDI1 protein) and the localization of the ubiquitination sites detected by mass spectrometry. Additionally, the diGly-modified peptides identified, within the ubiquitinated lysine marked in red, and the intensity values measured in each condition (WT, UBE3A^WT^ and LD, UBE3A^LD^) are also indicated. “∙” indicates that the ubiquitination site is not reported in PhosphoSitePlus database. DiGly peptides encompassing K133 and K161 were statistically more abundant upon UBE3A^WT^ overexpression [*t*-test, ^∗^*p*-value < 0.05, (mean ± S.E.M., *n* = 3)]. **(D)** UBE3A-dependent ubiquitination of three distinct DDI1-GFP mutants (K77R, K133R, or K161R) was monitored by western blot after isolating by GFP-pulldown. In red it is illustrated the ubiquitination pattern of DDI1, and in green the unmodified version of DDI1-GFP. Quantification and statistical analysis of the ubiquitination was performed with Image-J after normalizing FLAG intensities to GFP levels. Mutation on DDI1 lysine 133 significantly [one-way ANOVA, ^∗^*p*-value < 0.05, (mean ± S.E.M., *n* = 3)] abolishes its ubiquitination by UBE3A^WT^.

To infer DDI1 ubiquitination sites, MS-derived raw files corresponding to the slices of both mono- and poly-ubiquitinated DDI1 were jointly analyzed using MaxQuant software. Based both on MS/MS counts and LFQ intensity values, GFP-tagged DDI1 was, as expected, the most abundant protein detected by mass spectrometry (Figure 3A and Supplementary Table 1). The second most abundant protein was ubiquitin (Figure 3A) despite the lack of ectopic ubiquitin expression in this experiment; given the stringent washing conditions used in the pull-down protocol, it could be postulated that those ubiquitin moieties were covalently attached to DDI1. While the levels of DDI1 were practically the same independently of UBE3A activity, Ub levels were significantly increased in the sample transfected with UBE3A^WT^ (Figure 3B).

We detected numerous unmodified peptides that covered 76% of the DDI1-GFP sequence (Supplementary Figure 4A), but more interestingly we identified six diGly-modified peptides across the whole sequence of DDI1 (Figure 3C and Supplementary Table 2), and one on the *C*-terminally fused GFP protein (Supplementary Figure 4B). More precisely, we found that ubiquitin was conjugated to the following residues on DDI1: K77, which localizes in the *N*-terminal ubiquitin-like (UBL) domain; K133, K161 and K213 within the helical domain (HDD); K291, that resides in the aspartyl protease-like RVP domain; and K382, on the *C*-terminal end of the protein (Figure 3C). It is remarkable that besides K291 ubiquitination, whose homolog lysine had been previously reported to be ubiquitinated on experiments performed in rat brain (Na et al., 2012), the remaining five lysines detected in the present study (K77, K133, K161, K213, and K382) are novel ubiquitination sites of DDI1. Representative annotated spectra for these diGly modified peptides are shown on Supplementary Figure 4C.

Aiming to evaluate the effect of UBE3A on DDI1 ubiquitination, we compared the intensity of the diGly peptides between UBE3A^WT^ and UBE3A^LD^ overexpressing cells. The intensities of DDI1 ubiquitination sites K213-, K291-, and K382- containing diGly peptides were similar in both experimental conditions, indicating that UBE3A is not involved in modifying such resides (Figure 3C). On the contrary, the intensity of K133- and K161-bearing diGly peptides was induced 5- and 2- fold, respectively, in the UBE3A^WT^ overexpressing condition (Figure 3C), while total DDI1 levels remained constant (Figure 3B). Similarly, ubiquitination on lysine 77 also appeared to be enhanced upon UBE3A^WT^ overexpression, although this increase did not appear to be statistically significant, most probably due to the low MS/MS counts recorded (Supplementary Table 3). Overall, our data suggest that UBE3A may be involved in the ubiquitination of three (K77, K133, and K161) out of the six ubiquitination sites detected on DDI1.

To confirm that indeed UBE3A is responsible for ubiquitinating DDI1 on K77, K131, and K161, we generated three DDI1 point mutations, each with one of the above-mentioned lysines replaced by arginine. Following the same GFP pull-down procedure mentioned above, we isolated the DDI1-GFP mutants and monitored their ubiquitination by immunoblotting. As shown in Figure 3D, DDI1 ubiquitination upon UBE3A^WT^ overexpression was still observed for the K77R and K161R mutants to similar level as for wild type DDI1. Having detected by MS in wild type DDI1 a moderate increase of ubiquitination at those two lysines, their removal did, however, not prevent UBE3A to still ubiquitinate DDI1, probably due to the ubiquitination being transferred to an alternative lysine. By contrast, UBE3A-dependent DDI1 ubiquitination was significantly reduced in the case of the K133R mutant (Figure 3D and Supplementary Figure 4D). It must be noted that position K133 was the one at which the highest ubiquitinated peptide intensity increase had been observed by MS upon UBE3A^WT^ co-expression. In fact, the ubiquitination pattern of K133R co-expressed with UBE3A^WT^ was very similar to the pattern exhibited by wild type DDI1 co-expressed with UBE3A^LD^. These results indicate that UBE3A is responsible for ubiquitinating DDI1 on K133, and that the presence of this lysine 133 is necessary for UBE3A-mediated ubiquitination of DDI1.

### UBE3A-Dependent Ubiquitin Linkage Types on DDI1

**FIGURE 4:**
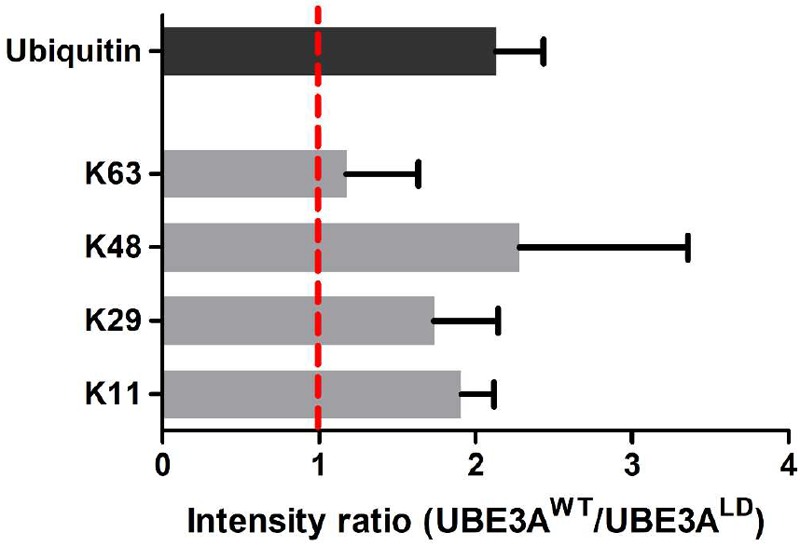
Analysis of the ubiquitin linkages formed in DDI1. Four ubiquitination sites on ubiquitin were confidently identified and quantified by mass spectrometry. The intensity ratios for each ubiquitin chain type are given as the ratio of the UBE3A^WT^ overexpressing condition over the intensity recorded for the UBE3A^LD^ sample. The intensity ratio for ubiquitin is also indicated for reference. As K29 was not detected in neither of the UBE3A^LD^ samples, its missing values were substituted by the lowest intensity value found in each replica.

Next, we studied the types of ubiquitin linkage formed on poly-ubiquitinated DDI1. For that purpose, we focused on the diGly peptides corresponding to ubiquitin within the gel slice containing poly-ubiquitinated DDI1-GFP (Figure 2). In line with previous results (Figure 1, 3B), the ubiquitin levels in this slice were also more abundant upon overexpression of UBE3A^WT^, and the ubiquitin intensity ratio between UBE3A^WT^ and UBE3A^LD^ samples displayed an approximately two-fold increase (Figure 4). MaxQuant analysis of this slice confidently detected four ubiquitination sites within ubiquitin: K11, K29, K48, and K63. The levels of K63 chains remained unaffected upon overexpression of UBE3A^WT^. K29 ubiquitin linkages appeared to be increased in the presence of UBE3A^WT^, in line with some earlier reports for UBE3A and other HECT E3 ligases (Chastagner et al., 2006; Xu et al., 2018), but we should note that those linkages were detected with very few counts and overlapped with a possible ubiquitination event also in K27 (Supplementary Table 3). More convincingly, the average from the three biological replicates analyzed displayed a clear increase of approximately two fold for both K48 and K11 chains in the UBE3A^WT^ over-expressing cells with respect to the control sample (Figure 4 and Supplementary Table 3). It is worth noting that the intensity fold change measured for the K48 and K11 diGly peptides were similar to the global fold-change observed for ubiquitin itself. Based on this data, and in partial agreement with earlier reports (Kim and Huibregtse, 2009), we conclude that UBE3A^WT^ overexpression indeed induces both K48 and K11 ubiquitin chains on DDI1.

### Identification of USP9X as the DUB Responsible for Deubiquitination of Human DDI1

**FIGURE 5:**
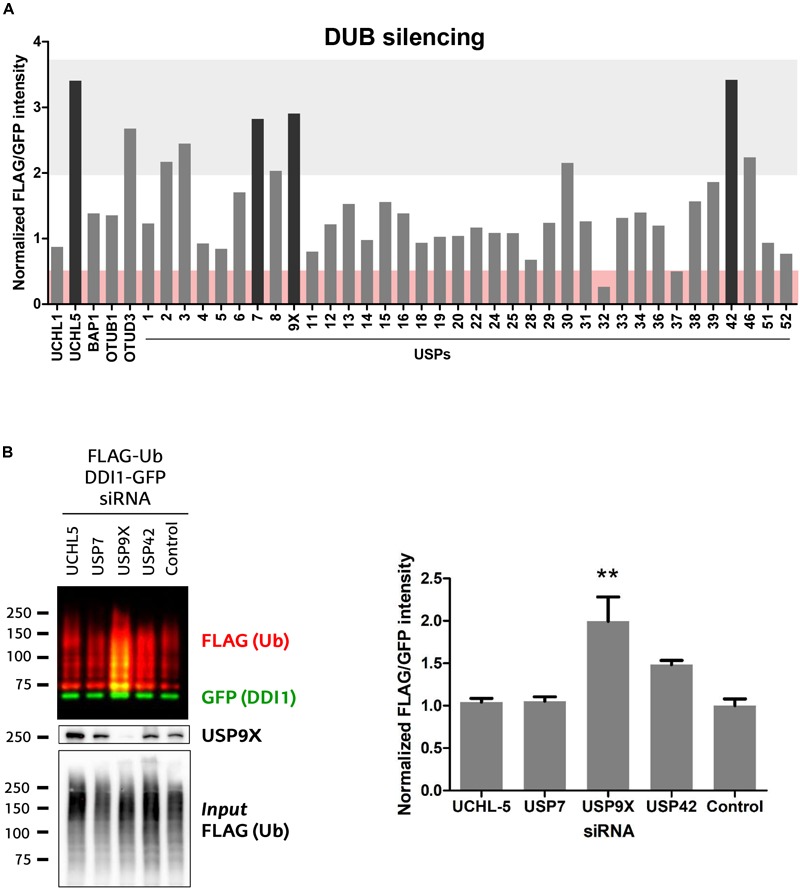
Screen of 41 human DUBs indicates USP9X to be the counteracting DUB of UBE3A. **(A)** 41 different human DUBs were silenced using 10 nM siRNAi. After GFP-pulldown of DDI1-GFP, FLAG/GFP intensity ratios were determined. Those ratios were normalized to the average of three lowest values on each 6-well experimental dataset. Upon silencing of most DUBs, the ubiquitination of DDI1 did not change quantitatively (0.5 < fold-change < 2, white). However, silencing of some DUBs decreased DDI1 ubiquitination (fold-change < 0.5, red), while some others increased it (fold-change > 2, gray). Black bars highlight the DUBs that affect more severely DDI1 ubiquitination (UCHL-5, USP7, USP9X, and USP42). **(B)** siRNAs were used to silence UCHL-5, USP7, USP9X, and USP42 DUBs and scramble siRNAi was used as a control. After GFP-pulldown, DDI1 ubiquitination was detected by western blot analysis using anti-FLAG (red) and anti-GFP (green) antibodies. Equivalent amounts of ectopically overexpressed ubiquitin was detected in the cell lysates [*Input* FLAG-(Ub)], while USP9X silencing was corroborated by anti-USP9X antibody (USP9X). Quantification was performed with Image-J after normalizing FLAG/GFP intensities. USP9X inhibition significantly enhanced DDI1 ubiquitination [one-way ANOVA, ^∗∗^*p*-value < 0.05, (mean ± S.E.M., *n* = 3)]. DDI1 ubiquitination also increased upon USP42 silencing, but not significantly, while no changes were observed for the UCHL-5 and USP7 experiments.

The ubiquitination state of any given protein is determined by the balance between the conjugating action of E3 ubiquitin ligases and their counteracting deubiquitinating (DUB) enzymes. Having demonstrated that UBE3A ubiquitinates DDI1, we next aimed to discover the DUB capable of deubiquitinating it. We screened an RNAi library in order to identify the DUB whose silencing would enhance DDI1 ubiquitination. For that purpose, we used HEK293T cells expressing DDI1-GFP and FLAG-Ub, in which 41 different DUBs were silenced individually. More precisely, we silenced 36 members of the USP family, as well as 3 and 2 members, respectively, of the UCH and OTU families (Figure 5A). To measure DDI1 ubiquitination, GFP-tagged DDI1 was first isolated by GFP pull-down, and then ubiquitin was detected by immunoblot with an anti-FLAG antibody. Changes in DDI1 ubiquitination were measured by normalizing the FLAG-Ub signal for each sample against its own GFP signal. Additionally, aiming to compare the results obtained in the several 6-well plate based experiments, we normalized this ratio to the average of the 3 lowest ratios on each dataset of 6 silencing experiments. As shown in Figure 5A, silencing of most DUBs did not substantially modify the ubiquitination state of DDI1 (normalized GFP/FLAG intensity between 0.5–2). Interestingly, upon silencing of UCHL-5, OTUD3 and 8 USP family members (USP2, 3, 7, 8, 9X, 30, 42, and 46) ubiquitination of DDI1 was quantitatively enhanced (normalized GFP/FLAG intensity > 2). From those 10 candidates, we focused our attention on the four DUBs having the greatest influence on DDI1 ubiquination: UCHL-5, USP7, USP9X, and USP42.

We then silenced the above mentioned four DUBs separately, and used a scramble siRNA as a control for this validation experiment that was performed in triplicate (Supplementary Figure 5A). The silencing of DUBs – UCHL-5, USP7, USP9X and USP42 – was confirmed by immunoblotting (Supplementary Figure 5B). After DDI1-GFP and FLAG-Ub overexpression in HEK293T cells, DDI1-GFP was isolated by GFP-pulldown, and the ubiquitination of DDI1 monitored by immunoblotting. As shown in Figure 5B, silencing of neither USP7 nor UCHL-5 had any influence on the ubiquitination state of DDI1. Downregulation of USP42, on the other hand, showed an increase on DDI1 ubiquitination, but this increase resulted to be statistically no significant. Importantly, this second round of screening clearly demonstrated that USP9X silencing results in enhanced DDI1 ubiquitination, and hence confirms that USP9X is required for deubiquitinating DDI1 (Figure 5B).

**FIGURE 6:**
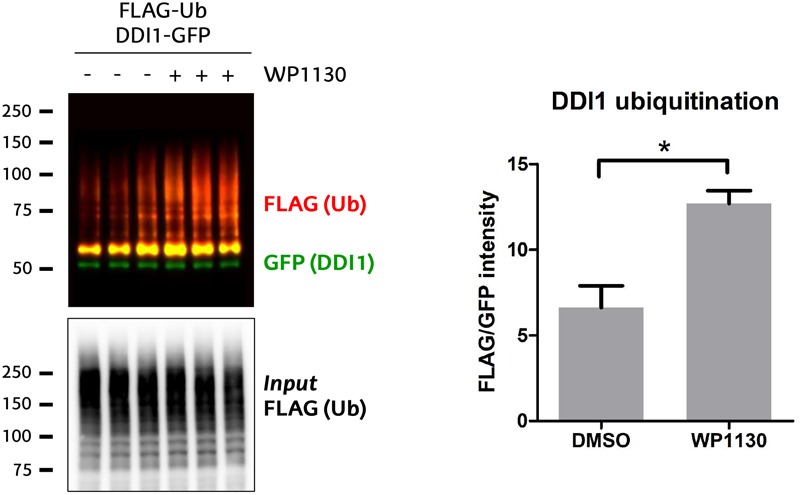
USP9X inhibition by WP1130 enhances DDI1 ubiquitination. Cells expressing GFP-DDI1 and FLAG-Ub were treated with 5 μM WP1130 or DMSO for 1 h. GFP-DDI1 was pulled down and its ubiquitination monitored by western blot. Anti-GFP antibody was used to detect unmodified GFP-DDI1 levels whereas anti-FLAG antibody was used to detect ubiquitination. The signal of FLAG was normalized to the GFP signal. Equivalent amount of transfected ubiquitin was detected in the cell lysates by measuring FLAG-Ub levels [*Input* FLAG-(Ub)]. Quantification using Image-J showed a statistically significant increase in DDI1 ubiquitination after WP1130 treatment [*t*-test, ^∗^*p*-value < 0.05, (mean ± S.E.M., *n* = 3)].

DUB inhibition has been considered as a potential therapeutic strategy for those diseases on which the function of a given E3 ligase is lost, and certain essential substrates are ubiquitinated at levels below their physiological requirement. A compound termed WP1130 has been previously used to experimentally inhibit USP9X (Kapuria et al., 2010; Sun et al., 2011), even if it is known that it also targets other DUBs (Kapuria et al., 2010). Since highly selective USP9X inhibitors are not yet available, we tested whether WP1130 would –presumably through USP9X– enhance the ubiquitination of DDI1. For that purpose, HEK293T cells were incubated for 1 h with 5 μM WP1130 or with DMSO as control. We then checked the ubiquitination pattern of GFP-DDI1 by immunoblotting, using the same GFP-pulldown protocol described above. Ubiquitination of DDI1 was significantly enhanced in cells treated with USP9X inhibitor WP1130 (Figure 6), supporting the earlier observations that DDI1 is an USP9X-regulated substrate.

Overall, our data indicate that UBE3A and USP9X are the E3 ligase and DUB, respectively, involved in modulating the ubiquitination of DDI1.

## Discussion

Poly-ubiquitinated proteins targeted for degradation might be recognized directly by proteasomal receptors or by proteasomal shuttling proteins. The first shuttling proteins – Ddi1, Rad23 and Dsk2 – were identified and characterized in *Saccharomyces cerevisiae* (Lambertson et al., 1999; Kaplun et al., 2005). Proteasomal shuttles contain an *N*-terminal ubiquitin-like (UBL) domain that interacts with the 26S proteasome (Finley, 2009), and a *C*-terminal ubiquitin-binding domain domain (UBD) that binds to ubiquitin or poly-ubiquitin chains (Bertolaet et al., 2001). When ubiquitinated, substrates are captured by the UBD domain, whereas the UBL domain binds to UBL receptors of the proteasome, delivering the substrates for degradation (Finley, 2009). Yeast Ddi1 and Ddi1-like shuttling proteins (e.g., Ddi2) show unique properties among canonical proteasomal shuttles. On the one hand, their UBL domain is able to bind ubiquitin (Nowicka et al., 2015), and despite having a ubiquitin-like fold, it does not interact or very weakly interacts with typical UBL receptors, including Rpn10 (Zhang et al., 2009) and Rpn1 (Rosenzweig et al., 2012). On the other hand, human DDI1 and Ddi1-like proteins from vertebrates lack the UBA domain (a type of UBD), but instead interact with ubiquitin through their UBL domain (Nowicka et al., 2015) or the ubiquitin-interacting motif (UIM) in the case of Ddi1-like proteins (Nowicka et al., 2015; Sivá et al., 2016). In line with this, a novel proteasomal shuttling mechanism has been proposed for yeast Ddi1 (Nowicka et al., 2015). Furthermore, Ddi1 and Ddi1-like proteins contain an additional domain called the retroviral protease-like (RVP) domain (Sirkis et al., 2006), making it very likely that besides their role in targeting substrates for proteasomal degradation, they may exert additional roles in the cell. Thus, further studies are necessary to fully understand the shuttling mechanism mediated by DDI1, but also to discover in which other cellular functions is involved.

Post-translation modifications (PTMs), including protein ubiquitination and phosphorylation, play a critical role in essentially all cellular processes by regulating the activity, localization or interactions of proteins. For example, it has been shown that the interaction between the proteasome and the proteasomal shuttle Rad23 is regulated by a phosphorylation occurring on the UBL domain of Rad23 (Liang et al., 2014, 23). In a recent study, we uncovered that human DDI1 is a substrate of the E3 ubiquitin ligase UBE3A, and as a consequence is ubiquitinated (Ramirez et al., 2018). Therefore, and bearing in mind the relevance of PTMs, we postulated that studying how DDI1 is ubiquitinated, or more precisely, identifying the DDI1 ubiquitination sites and chain types, may shed light into its regulation and the mechanism by which this proteasomal shuttle targets proteins to degradation. A recent large-scale proteomic study has reported that human DDI1 is ubiquitinated in K345 and K346, which reside in the RVP domain (Akimov et al., 2018). Moreover, studies in rat brain revealed that rat DDI1 K297 is also ubiquitinated (Na et al., 2012). In line with these studies, we here present the first evidence indicating that its human homolog DDI1 K291, which is also located in the RVP domain, is also ubiquitinated. As mentioned above, the function of the RVP domain, which is only present in Ddi1 and Ddi1-like proteins, is obscure. Therefore, the recent findings regarding ubiquitination events occurring on human DDI1 RVP domain might provide a baseline for future studies on deciphering the role of this unique domain.

Furthermore, our mass spectrometry-based approach revealed five novel ubiquitination sites in human DDI1: K77 on the UBL domain, K133, K161 and K213 in the HDD domain and K382 in the *C*-terminal of the protein. It is likely that ubiquitination of different residues on specific domains may affect differentially DDI1 function. Hence, future work will be necessary to elucidate the biological significance of each modification.

We are interested in elucidating the molecular mechanisms underlying Angelman syndrome, a rare neurological disease caused by the lack of functional E3 ubiquitin ligase UBE3A in the brain. For that reason, among the six DDI1 ubiquitination sites detected in the present study, our interest focused on the ones mediated by UBE3A, as it is likely that UBE3A-dependent ubiquitination events are altered in Angelman syndrome patients. Comparing the intensity of the diGly-modified peptides corresponding to DDI1 in samples overexpressing UBE3A^WT^ and UBE3A^LD^, we found that peptides bearing ubiquitinated K77, K133 and K161 appeared more abundant upon overexpression of UBE3A^WT^. Altogether, these results pointed to the N-terminal region of DDI1, including the ubiquitin-like domain and the double helical domain (HDD), as a ubiquitination hotspot region, where UBE3A mostly acts.

Out of the three putative UBE3A-dependent DDI1 ubiquitination sites detected, K133- and K161-containing peptides reached statistical significance. However, to further corroborate previous results, DDI1 K77, K133 and K161 were individually mutated to arginines. UBE3A-dependent ubiquitination of DDI1 was only significantly reduced when residue K133 was mutated, indicating that undoubtedly UBE3A is responsible for modifying this residue. It has been postulated that the HDD domain, which contains the K133 residue, may play a role in substrate recognition (Trempe et al., 2016). Therefore, it is plausible that UBE3A-dependent ubiquitination of DDI1 K133 has an influence on such recognition.

The fate of an ubiquitinated protein does not only depend on the specific residue that is modified, but also on the type of ubiquitin linkage that is formed in such residue (Swatek and Komander, 2016). A large variety of ubiquitin linkages differentially modulate proteins and their function in multiple cellular processes. Thus, we investigated using a high stringency pull-down protocol the ubiquitin chain types specifically and covalently linked to DDI1. In parallel to the overall increase in ubiquitin signal observed upon UBE3A^WT^ overexpression, a detailed MS-based analysis of the DDI1 ubiquitinated fraction revealed K48 and K11 as the chain types most prominently induced by overexpression of this E3 ligase, suggesting that the increase in ubiquitin signal most likely corresponds to the formation of those chain types.

K48-linked ubiquitin chains have been widely described to direct proteins to degradation via the proteasome (Finley, 2009; Grice and Nathan, 2016). Indeed, several studies have shown that UBE3A forms K48-type linkages, and consequently, modified proteins are predicted to be targeted for degradation (Wang and Pickart, 2005). K11 chains also improve the signal for proteasomal degradation, as evident from the fact that branched K11-linked chains formed by the anaphase-promoting complex (APC/C) increase the efficiency of proteasomal substrate recognition (Meyer and Rape, 2014). However, and in agreement with our previous work (Ramirez et al., 2018), the unchanged GFP signal on the various western blots (Figure 1, 3D) clearly indicates that UBE3A-dependent ubiquitination of DDI1 does not target this shuttling protein to degradation. One plausible explanation could be that this ubiquitination event leads to a neutralization of this proteasomal shuttle. If the role of DDI1 is to transport K48- or K11-modified proteins to the proteasome, when the client is the shuttle itself, shuttling might be interrupted, by blocking both the proteasomal interaction, as well as the recruitment of further clients. This UBE3A-mediated ubiquitination of DDI1 could thus explain our previous observations that overall proteasomal function is somehow dependent on UBE3A (Lee et al., 2014; Ramirez et al., 2018). Further analysis to decipher the function of K48 and K11 ubiquitin chains in DDI1 *in vivo* is likely to provide more light in this issue.

Most PTMs, including ubiquitination, are reversible modifications modulated by the co-ordinated action of two opposing enzymes: one adding a given chemical group to the protein, the other one removing it. In particular, protein ubiquitination is modulated by E3/DUB pairs. The regulation of the enzymes modulating protein PTMs is crucial for maintaining the appropriate balance of protein modification required for cellular homeostasis. In fact, as already mentioned, deregulation of these enzymes is implicated in a number of diseases, including cancer and rare neurological diseases (Ruprecht and Lemeer, 2014; Osinalde et al., 2018). Therefore, emerging therapeutic strategies are focused on the design of drugs that modify the biological action of these enzymes, and consequently restore appropriate cellular PTM levels. Indeed, in the past two decades, kinase inhibitors have been successfully used to treat cancer and other diseases (Bhullar et al., 2018). At present, great efforts are being made to develop similar strategies to treat diseases caused by altered protein ubiquitination. More precisely, DUBs are emerging as druggable targets for different diseases. Attempts have been done to inhibit DUBs in cancer therapy (D’Arcy et al., 2015), aiming to increase ubiquitination, and subsequent degradation, of substrates (Pinto-Fernandez and Kessler, 2016). Here we propose that modulating protein ubiquitination may represent a therapeutic approach for Angelman syndrome patients that lack UBE3A activity in the brain. More precisely, inhibition of the DUB counteracting the action of the E3 ligase UBE3A may help restoring the non-pathological ubiquitination levels of UBE3A substrates in Angelman syndrome patients.

So far, there are few studies showing that E3/DUB pairs act in a highly co-ordinated manner to regulate the ubiquitination and, hence, the activity of common substrates (Kee et al., 2005; Xie et al., 2013; Bingol et al., 2014; Sharma et al., 2014). It has been shown that Ubp2 antagonizes Rsp5 E3 ligase activity by forming a complex together with Rsp5 and Rup1 that deubquitinate Rsp5 substrates (Kee et al., 2005). Additionally, the E3/DUB pair UBR5/DUBA acts to modulate the production of IL-17 in T-cells (Rutz et al., 2015). Nevertheless, the identity of the DUB antagonizing the action of UBE3A has remained elusive, so far. Our study is pioneer in revealing USP9X as the DUB counteracting UBE3A. We demonstrate that siRNA-directed USP9X silencing, as well as treatment with WP1130 (a previously used partially specific USP9x inhibitor) significantly enhance human DDI1 ubiquitination. While it is true that the partial specificity of WP11330 could confound the obtained results, interestingly, USP9X has been earlier reported to be involved in X-linked intellectual disability (Homan et al., 2014). Furthermore USP9X is also known to have roles on axonal growth and neuronal cell migration (Homan et al., 2014) as well as in the regulation of seizures (Paemka et al., 2015). USP9X is also known to deubiquitinate α-synuclein, which is central to the pathogenesis of Parkinson disease (PD) (Rott et al., 2011). It has been suggested that drugs that modulate the activity of USP9X, together with enhancers of autophagy or proteasomal activity, may help decrease the levels of α-synuclein and provide a novel therapeutic strategy to treat α-synucleinopathies. Similarly, based on our results, we propose that attenuating the activity of USP9X may contribute to enhance the ubiquitination levels of UBE3A substrates that might be decreased in Angelman syndrome patients.

## Author Contributions

NE, JAR, JMA, and UM designed the experiments. NE, JB, and KA performed the experiments. NE, NO, JR, BL, KA, and UM analyzed the experiments. NE, NO, and UM wrote the manuscript.

## Conflict of Interest Statement

The authors declare that the research was conducted in the absence of any commercial or financial relationships that could be construed as a potential conflict of interest.
