# Supplementary material for: Detailed Dissection of UBE3A-Mediated DDI1 Ubiquitination
Source: Front Physiol. 2019 May 3;10:534. doi: 10.3389/fphys.2019.00534 (PMC6509411; doi:10.3389/fphys.2019.00534)
Supplement: Supplementary file 4 [file Data_Sheet_1.docx]

Supplementary Material

# Supplementary Figures and Tables

## Supplementary Figures

**
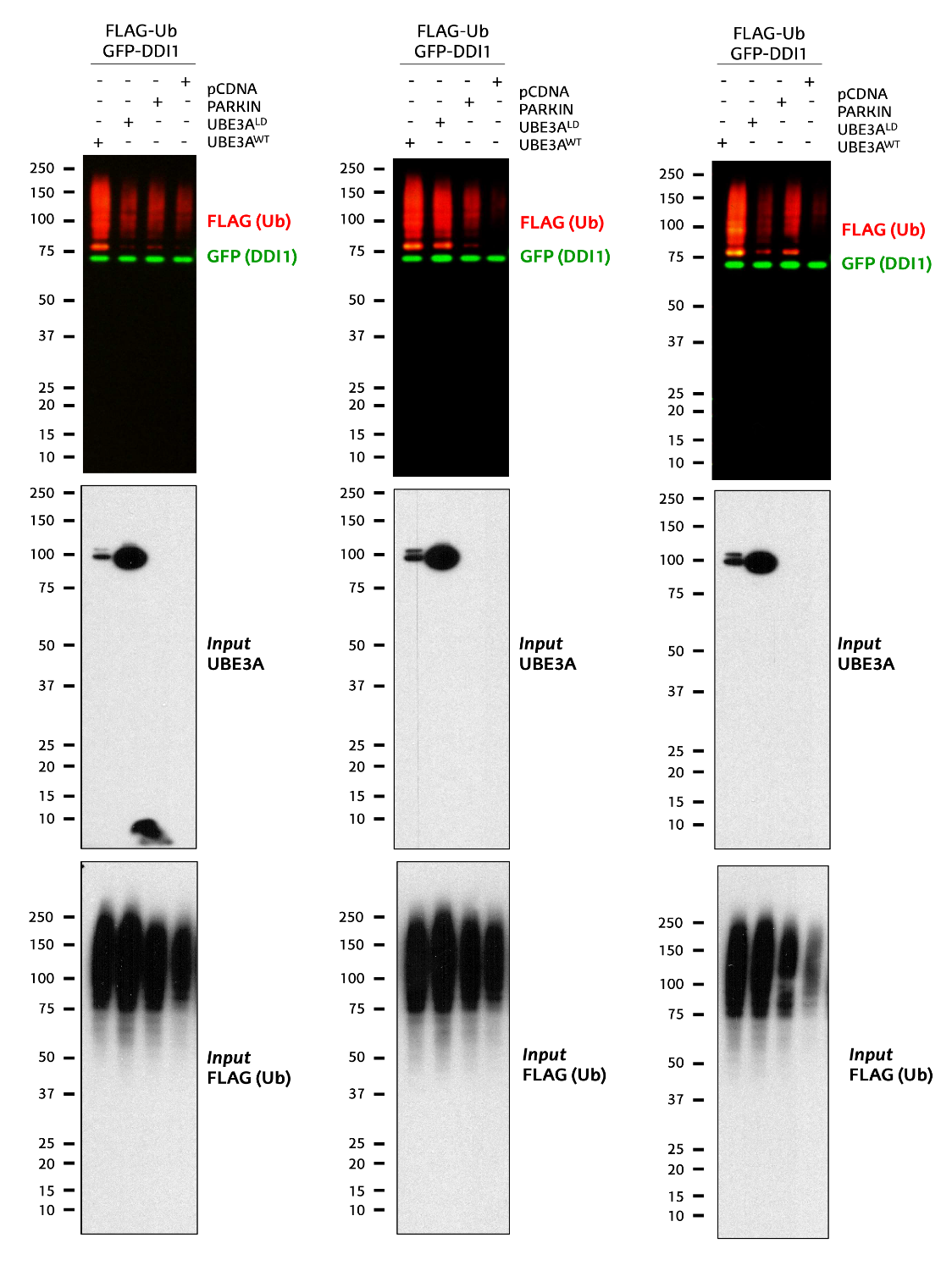
**

**Supplementary Figure 1. UBE3A ubiquitinates human DDI1 in HEK293T cells.** GFP-DDI1 ubiquitination upon wild type UBE3A (UBE3A^WT^), ligase dead UBE3A (UBE3A^LD^), Parkin E3 ligase (PARKIN) and control (pCDNA3.1) was detected by Western blot analysis in triplicate. Anti-FLAG antibody (red) was used to detect DDI1 ubiquitination, while the unmodified form of GFP-DDI1 was detected using anti-GFP antibody (green). UBE3A overexpression was assured in the whole cell lysates using anti-UBE3A antibody (*Input* UBE3A) while FLAG signal in the inputs corroborated equal amounts of material in all samples (*Input* FLAG-(Ub)). Overexpression of UBE3A^LD^, PARKIN and pCDNA3.1 did not affect DDI1 ubiquitination, while the presence of UBE3A^WT^ increased DDI1 ubiquitination levels.


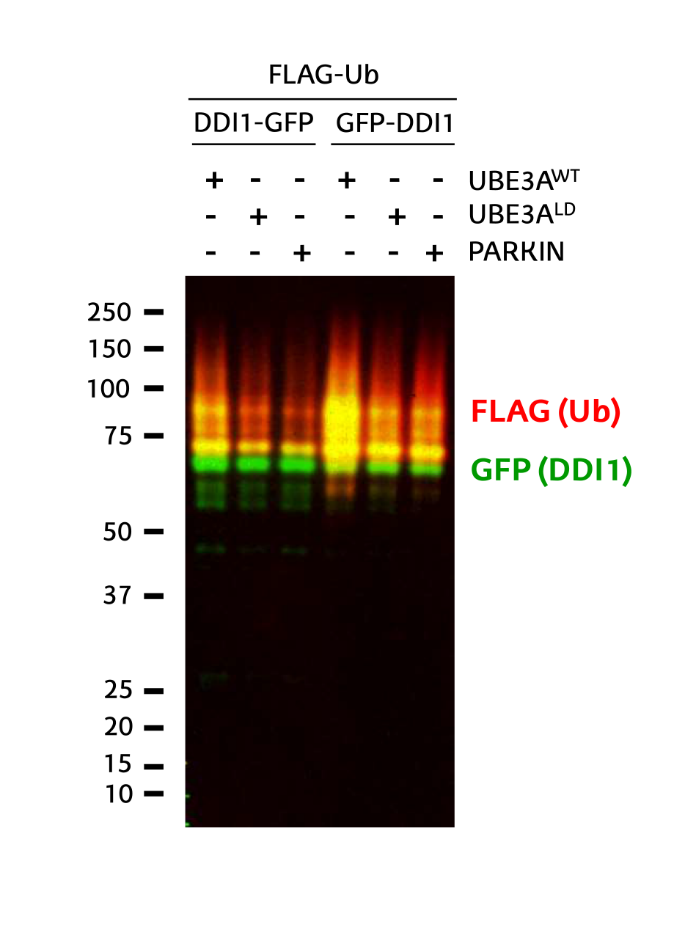


**Supplementary Figure 2. UBE3A-mediated DDI1 ubiquitination is independent from the location of the GFP tag.** N-terminally-tagged DDI1 (GFP-DDI1) and C-terminally tagged DDI1 (DDI1-GFP) were co-transfected with FLAG-tagged ubiquitin and three different E3 ligases: UBE3A^WT^, UBE3A^LD^ or PARKIN. Anti-GFP antibody was used to detect the GFP-tagged-unmodified DDI1 (green) and anti-FLAG antibody was used to detect ubiquitination (red). UBE3A overexpression clearly enhances DDI1 ubiquitination and this effect is independent of whether DDI1 is N- or C-terminally fused to GFP.


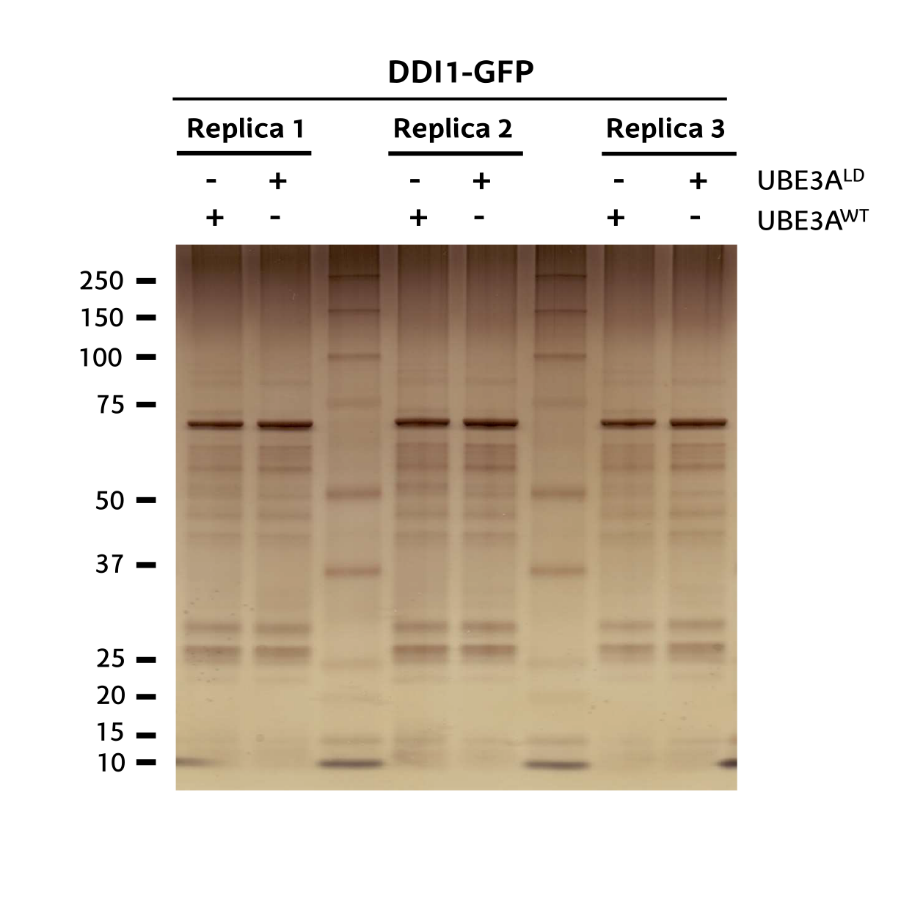


**Supplementary Figure 3. Silver staining of DDI1-GFP pulldown elutions.** The efficiency of GFP pull-downs was evaluated by silver staining after resolving by SDS-PAGE 10% of each neat elution. Gel bands visualized by SilverQuest kit showed a similar amount of material both among different samples (UBE3A^WT^ and UBE3A^LD^) as well as among different replicas.

**
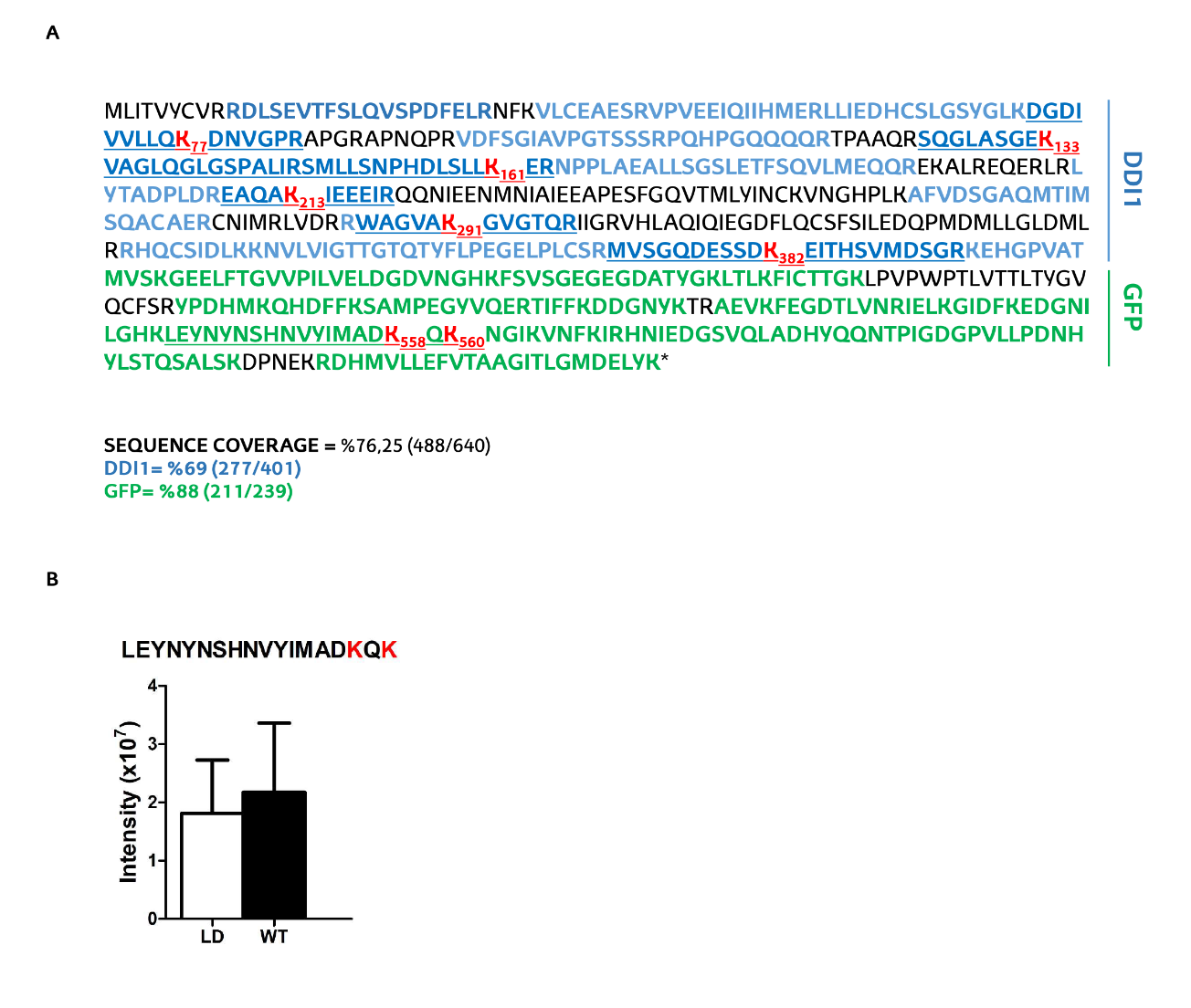
**

**
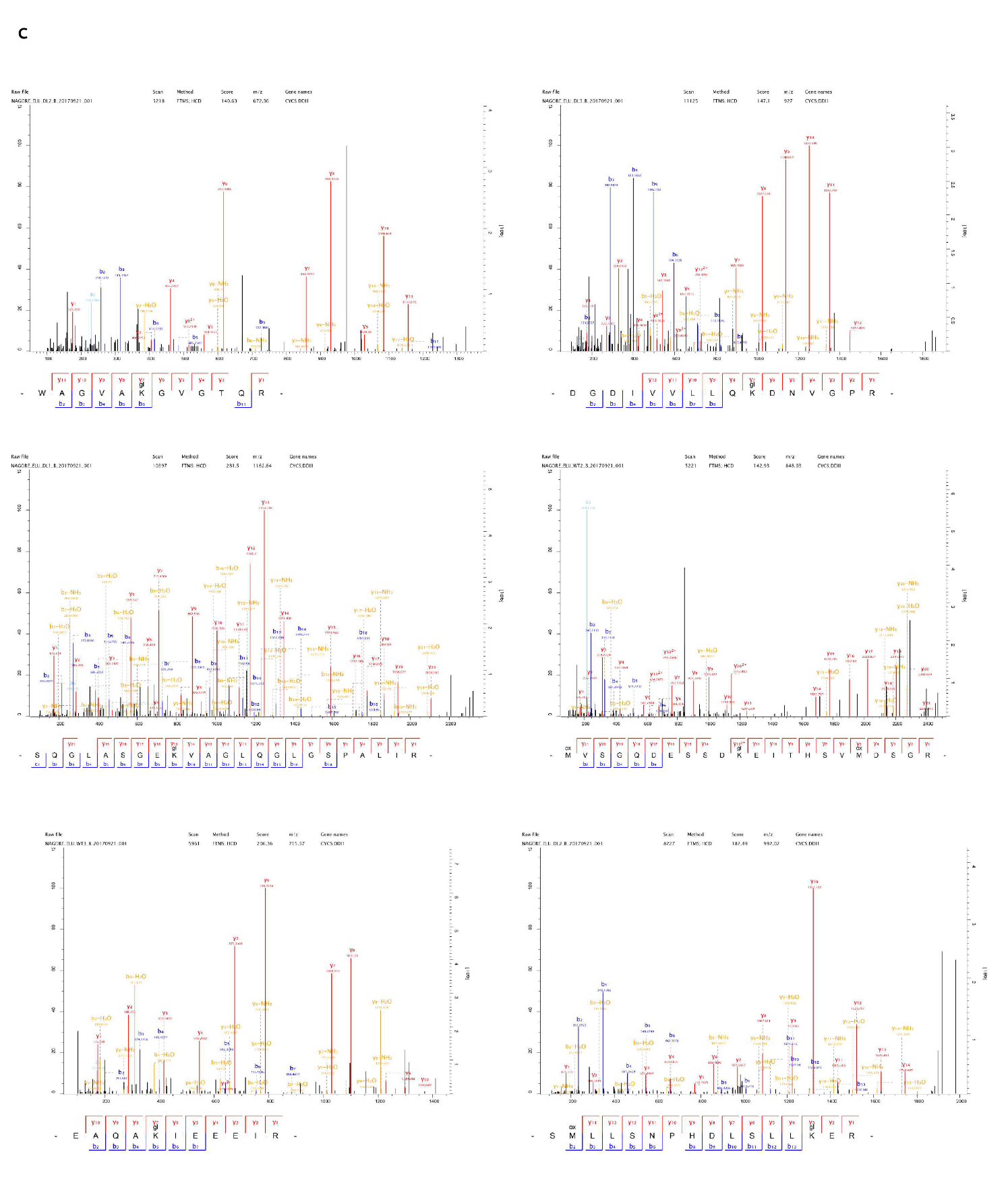

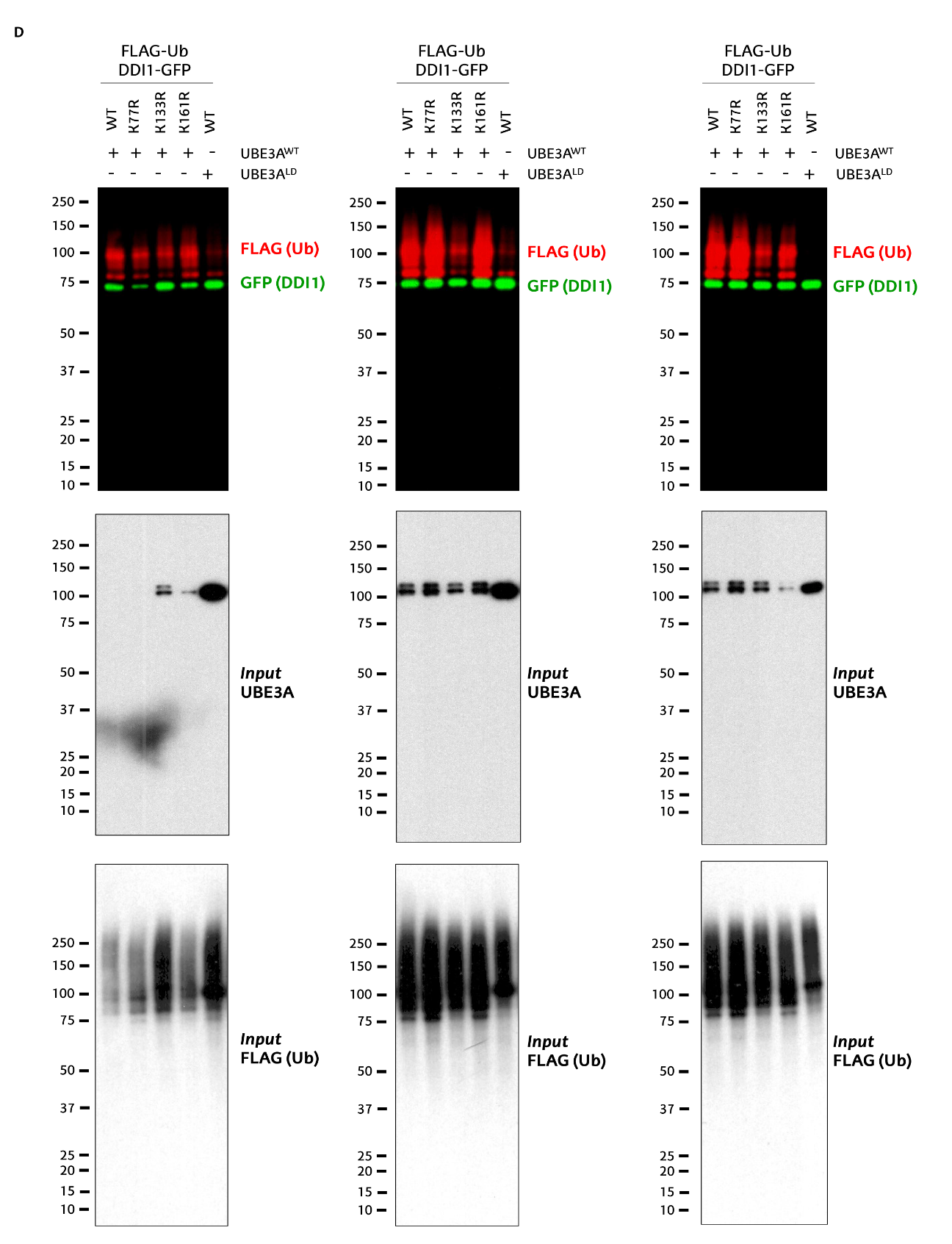
**

**Supplementary Figure 4. UBE3A ubiquitinates DDI1 on K133.** **A)** Sequence coverage for DDI1-GFP is shown, where peptides identified for DDI1 are highlighted in blue, and peptides identified for GFP in green. Underlined peptides correspond to ubiquitinated or diGly-modified peptides. In each case, the exact lysine that is modified is highlighted in red, and its position within the sequence is indicated. At the bottom, the percentage of sequence coverage obtained for DDI1-GFP, DDI1 and GFP are shown. **B)** The diGly-modified peptide identified in the GFP sequence is shown. In red are marked the two possible ubiquitinated lysines. Intensities upon UBE3^WT^ overexpression show no statistical differences in comparison to the UBE3A^LD^ sample. **C)** Representative annotated spectra for all DDI1 diGly modified peptides. **D)** Ubiquitination of different DDI1 mutants – wild type (WT), K77R, K133R, K161R – was detected by Western blot in triplicate upon wild type UBE3A (UBE3^WT^) and ligase dead UBE3A (UBE3A^LD^) overexpression. Anti-FLAG antibody (red) was used to detect ubiquitination, while anti-GFP antibody (green) detected the non-modified DDI1-GFP. UBE3A overexpression was corroborated by anti-UBE3A antibody (*Input* UBE3A) in the whole cell extract, and FLAG-ubiquitin was measured as loading control (*Input* Flag-(Ub)). Mutating DDI1 on lysine 133 completely abolishes its UBE3A-mediated ubiquitination.


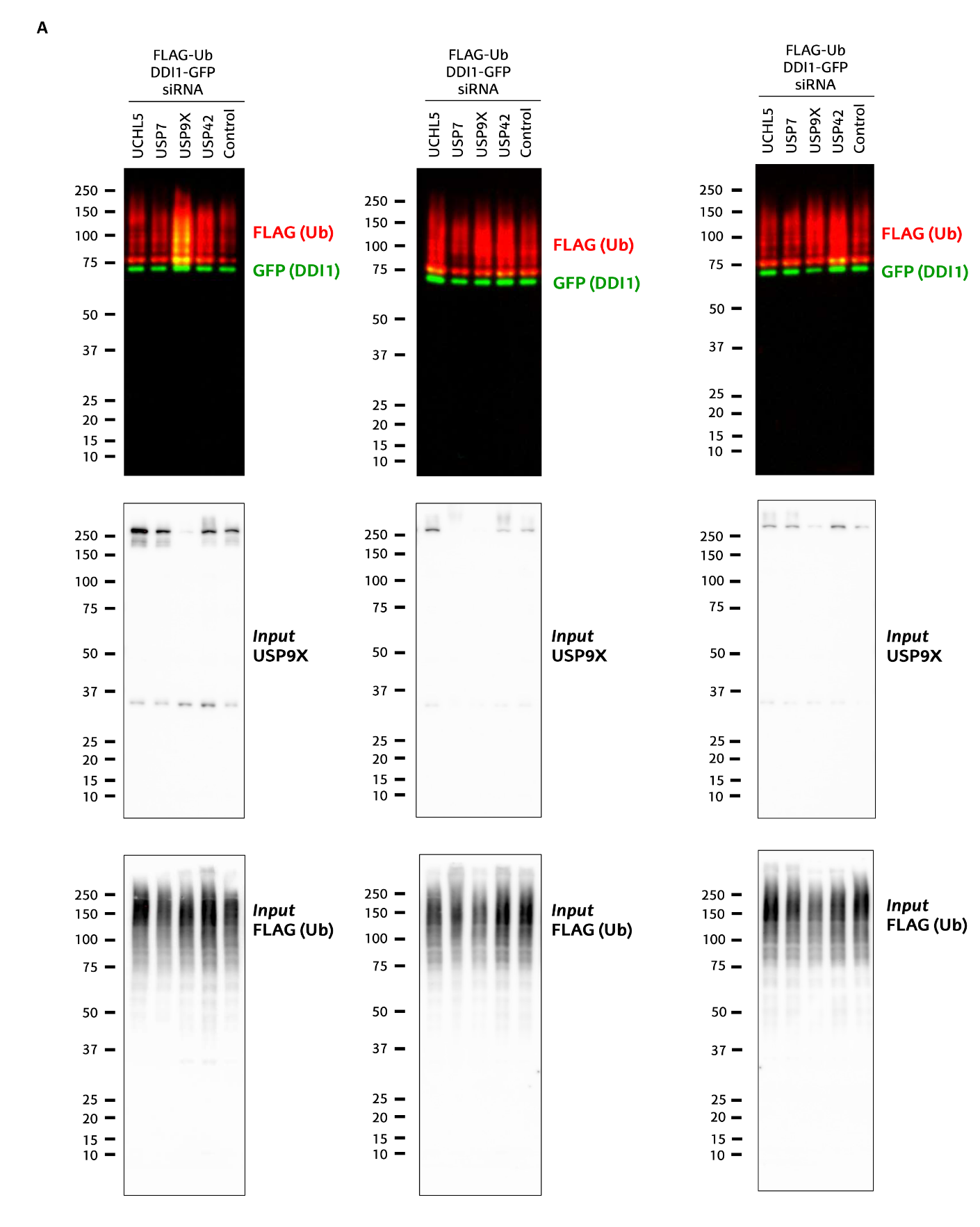


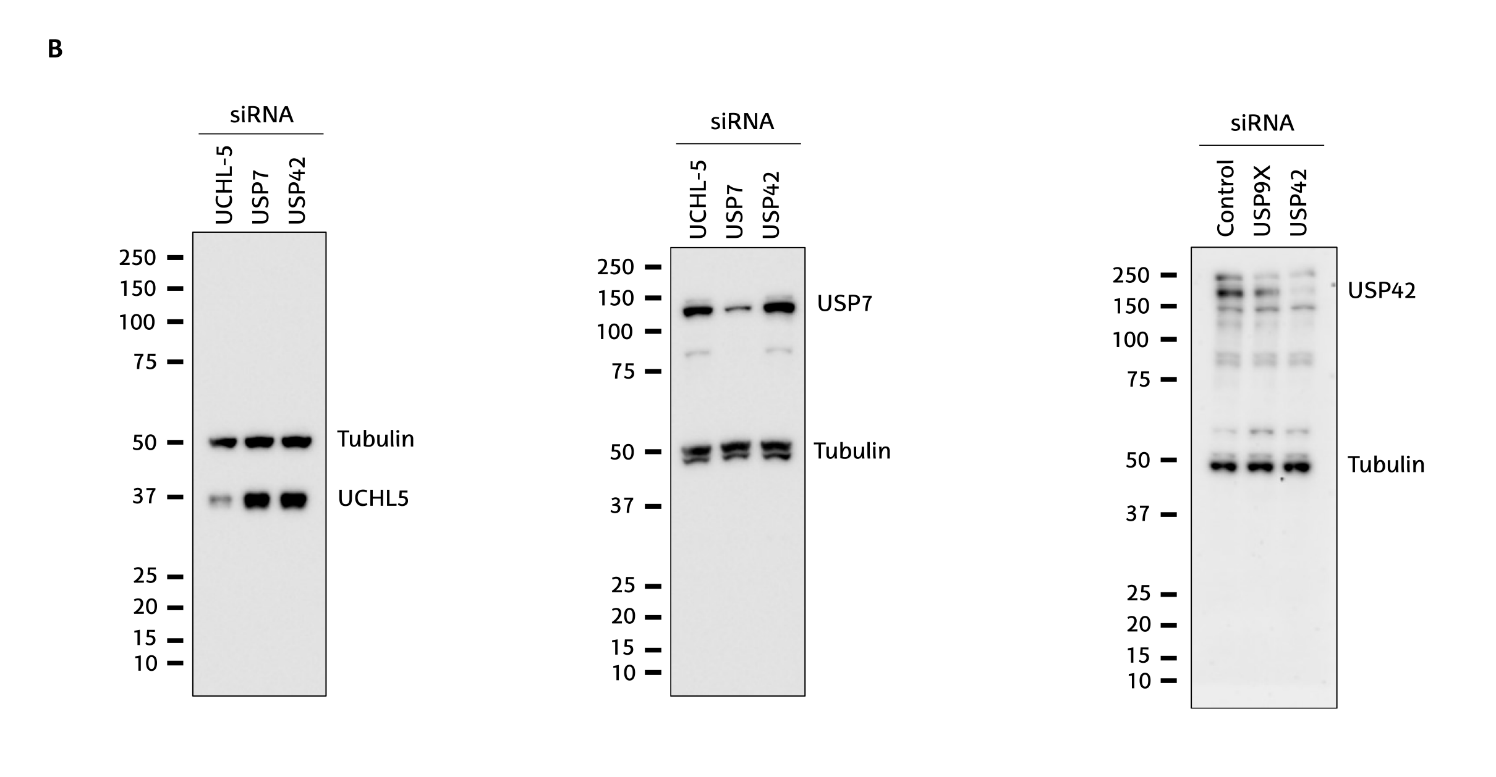


**Supplementary Figure 5. USP9X deubiquitinates DDI1, counteracting UBE3A function. A)** Different DUBs were silenced in triplicate – UCHL-5, USP7, USP9X, USP42 – using 10nM siRNAi and compared to the control sample containing scramble siRNAi. DDI1-GFP ubiquitination was detected by Western blot using anti-Flag antibody (red). Anti-GFP antibody (green) was used to detect the non-modified DDI1-GFP levels. Silencing of USP9X was corroborated in whole cell extract using anti-USP9X antibody (*Input* USP9X), while FLAG-ubiquitin was detected as loading control (*Input* FLAG-(Ub)). Silencing of UCHL-5, USP7 and USP42 did not affect DDI1 ubiquitination, while USP9X silencing increased it. **B)** Silencing of UCHL-5, USP7 and USP42 was further corroborated using their respective antibodies. All DUBs were silenced efficiently.

## Supplementary Tables

**Supplementary table 1. Proteins detected by mass spectrometry in the analysis of DDI1 ubiquitination sites and chain types.** MonoUb and polyUb band were jointly analysed.

**Supplementary table 2. List of the diGly modified peptides detected by mass spectrometry for the analysis of ubiquitination sites.** MonoUb and polyUb band were jointly analysed.

**Supplementary table 3. List of the diGly modified peptides detected by mass spectrometry for the analysis of chain types.** Poly-Ub slice was analysed only. In red, the missing values replaced for the K29 linkage.
